# Implementing people-centred health systems governance in 3 provinces and 11 districts of Afghanistan: a case study

**DOI:** 10.1186/1752-1505-9-2

**Published:** 2015-01-07

**Authors:** Zelaikha Anwari, Mahesh Shukla, Basir Ahmad Maseed, Ghulam Farooq Mukhlis Wardak, Sakhi Sardar, Javid Matin, Ghulam Sayed Rashed, Sayed Amin Hamedi, Hedayatullah Sahak, Abdul Hakim Aziz, Mariah Boyd-Boffa, Reshma Trasi

**Affiliations:** Leadership, Management and Governance Project - Afghanistan (LMG-AFG), Management Sciences for Health (MSH), House # 24, Darul Aman Road, Kabul, Afghanistan; Management Sciences for Health, 4301 N. Fairfax Drive, Suite 400, Arlington, VA 22203 USA; Wardak Provincial Public Health Office, Maidan Shahr, Afghanistan; Khost Provincial Public Health Office, Khost, Afghanistan; Herat Provincial Public Health Office, Herat, Afghanistan; Ministry of Public Health, Great Masoud Square, Kabul, Afghanistan; Center for Leadership and Management, Management Sciences for Health, Medford, MA USA

**Keywords:** Health, Governance, Health systems, Health systems governance, Health systems performance, Fragile and conflict affected environments

## Abstract

**Background:**

Previous studies show that health systems governance influences health system performance and health outcomes. However, there are few examples of how to implement and monitor good governing practices in fragile and conflict affected environments. Good governance has the potential to make the health system people-centered. More research is needed on implementing a people-centered governance approach in these environments.

**Case description:**

We piloted an intervention that placed a people-centred health systems governance approach in the hands of multi-stakeholder committees that govern provincial and district health systems. We report the results of this intervention from three provinces and eleven districts in Afghanistan over a six month period. This mixed-methods exploratory case study uses analysis of governance self-assessment scores, health management information system data on health system performance, and focus group discussions. The outcomes of interest are governance scores and health system performance indicators.

We document the application of a people-centred health systems governance conceptual model based on applying four effective governing practices: *cultivating accountability*, *engaging with stakeholders*, *setting a shared strategic direction*, and *stewarding resources responsibly.* We present a participatory approach where health system leaders identify and act on opportunities for making themselves and their health systems more accountable and responsive to the needs of the communities they serve.

**Discussion and evaluation:**

We found that health systems governance can be improved in fragile and conflict affected environments, and that consistent application of the effective governing practices is key to improving governance. Intervention was associated with a 20% increase in antenatal care visit rate in pilot provinces. Focus group discussions showed improvements across the four governing practices, including: establishment of new sub-committees that oversee financial transparency and governance, collaboration with diverse stakeholders, sharper focus on community health needs, more frequent presentation of service delivery data, and increased use of data for decision making.

**Conclusions:**

Our findings have implications for policy and practice within and beyond Afghanistan. Governance is central to making health systems responsive to the needs of people who access and provide services. We provide a practical approach to improving health systems governance in fragile and conflict affected environments.

**Electronic supplementary material:**

The online version of this article (doi:10.1186/1752-1505-9-2) contains supplementary material, which is available to authorized users.

## Background

Afghanistan, with the support of its development partners, has made great strides in reconstruction of its health system that was decimated by protracted periods of conflict which continues to this day [1, 2]. The country has made impressive gains in improving access to basic health services, increasing life expectancy, and reducing under-five mortality and maternal mortality. Leadership and management of its health system have improved and a robust health management information system is in place. Contracting of the delivery of basic package of health services with the non-government organizations, and balanced scorecard have been successfully and consistently applied [3–5]. Many governance challenges remain in the central ministry of public health and its offices in the provinces and districts, and hospitals and health facilities. While multi-stakeholder committees have been established in the provinces and districts, and consultative committees at health facility and village levels, they do not interact sufficiently with each other and for that matter, with the health facilities and communities. There is a lack of concerted action. Decision making processes are not adequately open and transparent, and these committees are not equipped with adequate skills, authority or resources to carry out their mandated governance functions. Intersectoral collaboration is scarce at all levels. Despite the challenges, these multi-stakeholder committees in the provinces and districts are an invaluable entry-point to the governance of the provincial and district health systems.

Recent studies demonstrate that good governance, especially at the decentralized levels, can improve health outcomes [6, 7]. Earlier research has shown that poor governance overall, and especially in the health sector, has contributed to poor health outcomes [8–12]. Despite being recognized as one of the essential building blocks of a health system, governance remains an obfuscated and inaccessible concept. Different conceptual frameworks have been proposed to define and measure governance, and its potential effect on health system performance and health service delivery [13–20]. While this diversity of frameworks helps understand governance as a construct in the context of health, these do not illustrate ways to apply effective governing practices in the fragile and conflict affected environments, which presents unique challenges and security risks to those trying to improve its governance. There is paucity of guidance in the literature for the practitioners in these environments who want to improve governance of their health systems on how to do it.

In this paper, we present the results of a pilot health systems governance intervention in three provinces and eleven districts in Afghanistan. At the central level, the Ministry of Public Health (MOPH) contracts non-government organizations to provide services through health posts and health facilities. At the provincial and district level, health coordination committees are given responsibility for monitoring and oversight of health service delivery.

The two research questions this study addresses are: 1) does a people-centred health systems governance intervention based on learning and applying a set of good governance practices improve the governance of provincial and district health systems in a fragile and conflict affected environment, and 2) if so, does improved governance result in better health system performance?

The pilot was conducted in four phases over a year. In the first phase, provincial and district health systems governance guides were drafted, based on the effective governing practices, in consultation and with participation of provincial and district health coordination committees. In the second phase, based on the guides, these committees explored opportunities to improve the governance of their provincial and district health systems, and designed a specific governance development action plan for this purpose. They also measured their governance at baseline using five self-assessment instruments. In the third phase, the committees implemented and monitored their action plans over a period of six months. In the fourth and final phase, the committees evaluated their implementation of the action plans, and measured their governance post-intervention using the same five self-assessment instruments.

We define governance as (1) setting a shared strategic direction and objectives; (2) making policies, laws, rules, regulations, or decisions, and raising and deploying resources to accomplish strategic goals and objectives; and (3) overseeing and making sure that the strategic goals and objectives are accomplished [21]. Governance is effective when strategic objectives are successfully and efficiently met, but good governance goes even further. Governance is good when (1) decisions are based on information, evidence, and shared values; (2) the process is transparent, inclusive, and responsive to the needs of the people, the ministry, or the organization that it serves; (3) those who make and those who implement decisions are accountable; (4) strategic objectives are effectively, efficiently, ethically, and equitably met; and (5) the vitality of the organization and the services it provides are sustained [21].

Recently, Barbazza and Tello reviewed previous efforts to define, describe, and operationalize the health governance function, and compared 19 definitions of governance in the context of health, including this definition [22]. They examined definitions available in the literature on 23 values, sub-functions, and outcomes. This definition covers 16 of them. The definitions differ in the degree to which they emphasize various dimensions of governance. Barbazza and Tello underline a need for a concerted effort toward a more accessible understanding of health governance that is both practical and actionable for policy-makers. We provide such an approach in this paper.

WHO calls a health system people-centred when it is rooted in principles of human rights and dignity, nondiscrimination, participation and empowerment, universal access and equity, and partnership [23, 24]. Similarly, perspectives from the field suggest that a health system is likelier to meet the health needs and expectations of people and communities and improve health outcomes when people who govern health systems - public or private - carry out activities and take actions to cultivate accountability; engage with stakeholders; set a shared strategic direction; steward resources responsibly to meet the health needs of the people; and invest in transparency, inclusion and participation, gender responsive policies, intersectoral collaboration, leadership development, measurement of performance including their own governance performance, and use of technology [25]. Our health systems governance intervention is largely based on these principles and practices.

### Conceptual framework

**Figure 1:**
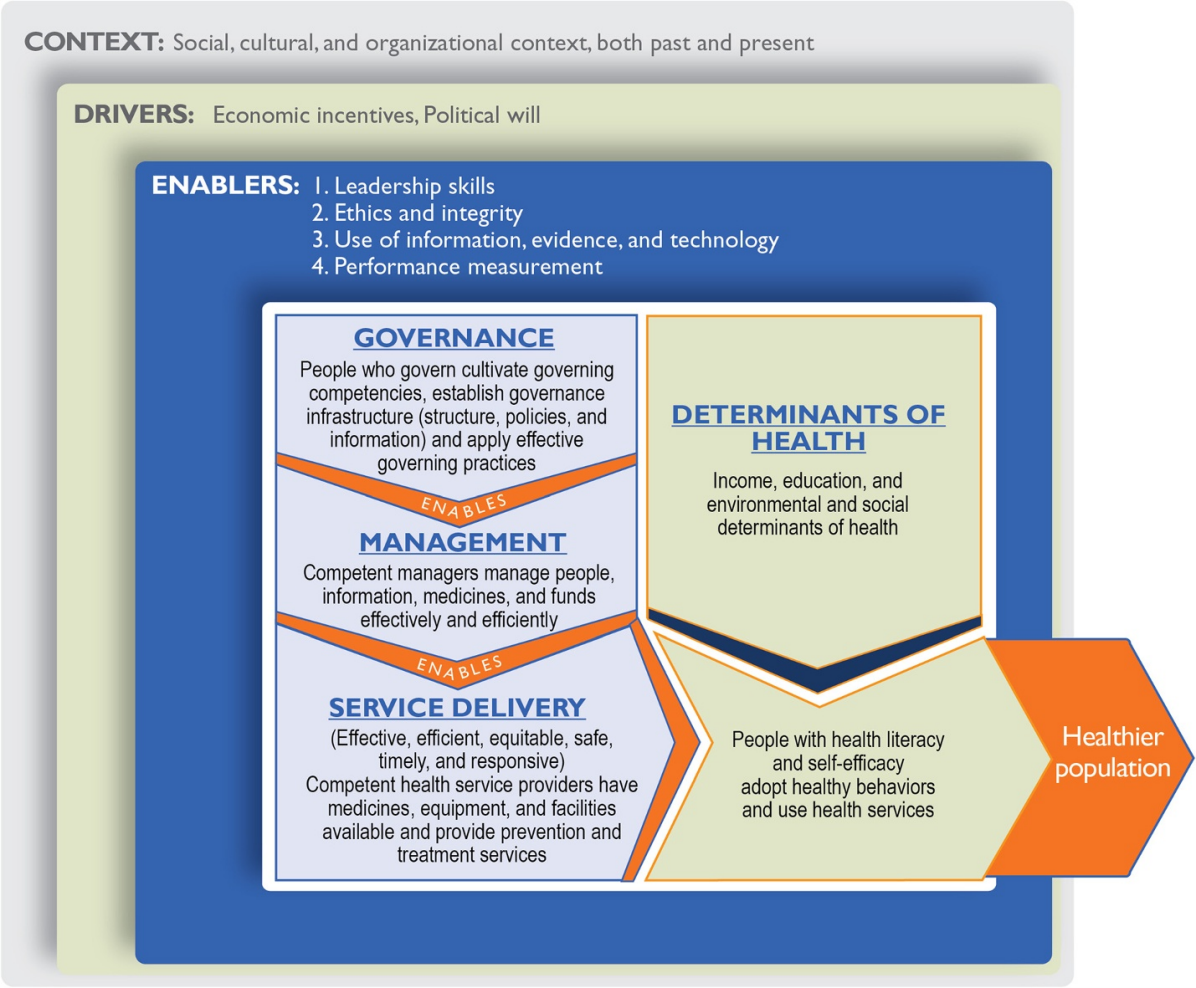
**Conceptual model of health systems governance.**

In an earlier unpublished work, we conducted a targeted literature review, a survey of 477 health leaders and managers in 80 low and middle income countries, and key stakeholder interviews of 25 health leaders in 16 countries to understand what makes governance effective in the context of health. After analyzing our findings, we concluded that four over-arching governing practices make governance effective in meeting the health needs and expectations of people and communities: *cultivating accountability*, *engaging with stakeholders*, *setting a shared strategic direction*, and *stewarding resources in a responsible way*. The fifth practice of periodically assessing governance and continuously trying to enhance it ensures that the four practices are consistently applied. The study also found good leadership facilitates effective governance, and that sound management sustains it. We developed our conceptual model of health systems governance based on the findings of this prior study (Figure 1). At the center of this model are people who govern, health managers, health providers, health workers, community leaders, and patients and health service users. We designed our four-phase pilot study based on this model.

This framework is applicable to both fragile and non-fragile contexts. Fragile contexts are characterized by a cycle of insecurity, poor governance, serious deficiencies in the delivery of public services, and mistrust – one feeding into the other, which makes it harder to govern well in these environments.

### Case description

#### Institutional context

In Afghanistan, *shuras* or informal consultative assemblies of elders have a long and well-established tradition of resolving disputes and solving contentious issues in communities. The Provincial Public Health Coordination Committee (PPHCC) is, in contrast, a formal multi-stakeholder committee with a set of distinct responsibilities established by the MOPH at provincial level in the early 2000s. PPHCCs have been functional since in all 34 provinces. PPHCCs provide a forum for coordination and information sharing among various stakeholders in the provincial health system. They discuss community health concerns, and coordinate and participate in all stages of the emergency response. They also monitor and supervise health posts and health facilities. They are expected to meet on a monthly basis and coordinate delivery of the basic package of health services, and the essential package of hospital services.

The MOPH has also formally established consultative community health shuras and health facility shuras at health post, health facility, and district hospital levels. Hospital community boards were established at the provincial hospital level. In the last 4 to 5 years, the MOPH has been establishing District Health Coordination Committees (DHCCs) in the districts to perform a role similar to that of the PPHCCs in the provinces.

The PPHCC is a multi-sectoral governing body chaired by the Provincial Public Health Director. It has 21 members that include nine appointed provincial public health officers, provincial hospital director, director of the Institute of Health Sciences, two representatives of NGOs providing health services at health post and health facility levels, two district health officers, and one representative from each of the following: ministry of women’s affairs, provincial reconstruction team, private health sector, elected provincial council, UNICEF, and WHO. Thirteen members have voting powers that include six officials from the provincial public health office; provincial hospital director; and members of the private health sector, provincial council, UNICEF, WHO, and NGOs. Decisions in the PPHCC are usually based on consensus. If there is no consensus, the decision is put to a vote. A decision requires a quorum and a majority of voting members in favor. The members are not paid compensation or sitting fees for serving on the PPHCC.

Similarly, the DHCC is chaired by the District Public Health Officer and its members include a district governor’s representative, private health sector representative, religious leader from the district, director of the district hospital, an implementing NGO representative, head of the district education department, and head of the district council which is an informal assembly of elders in the district. Decision making in DHCC is similar to that of PPHCC i.e. decisions are generally taken by consensus, and if it fails, by a majority vote. The PPHCCs, DHCCs, and community and facility health shuras are performing a governing role. PPHCC and DHCC governance has the potential to make a difference in the care delivered during patient visits at the health facilities.

## Methods

The study is a mixed-methods exploratory case study based on the analysis of governance self-assessment scores, health management information system data on health system performance, and focus group discussions. Governance scores measure governing practices of the provincial and district health coordination committees. The outcomes of interest were these governance scores and select health system performance indicators.

### First phase: participatory development of provincial and district health systems governance guides

PPHCC and DHCC governance guides were developed based on the effective governing practices through a consultative process consisting of surveys and workshops. A survey of key informants from 15 PPHCCs was conducted to perform situation analysis i.e. to learn how well PPHCC and DHCC committees and subcommittees have been performing their governance function, if and how their role could be expanded, who else could be invited to committee meetings, whether more subcommittees were needed, what principles members should adhere to, what responsibilities individual members should have, what competencies the members and chair should possess, and most importantly, how to make their governance more effective so the health needs and expectations of people and communities are met. Similar questions were discussed in a 3-day workshop with the Provincial Liaison Directorate of the MOPH which deals with the provincial and district offices of the ministry.

The MOPH selected the convenience sample of three PPHCCs (of Wardak, Khost and Herat provinces) and eleven DHCCs (of Narkh, Jalrez, Sayedabad, Ismailkhail-Mandozai, Alisher-Terezay, Qarabagh, Istalif, Eshkamish, Garmser, Spin Boldak and Qaysar districts) for the purpose of pilot testing (see Tables 1 and 2). Equal number of provinces and districts similar to pilot provinces and districts in terms of geographical location, cultural, ethnic, and economic profile, access to healthcare services, and security situation were selected for comparison purposes. Of eleven districts, five were from provinces where a province-level intervention also took place, and the remaining six were from provinces where there was no province-level interventions.

**Table 1:** **Pilot and comparison provinces**

No.	Intervention provinces	Comparison provinces
1	Wardak	Ghazni
2	Khost	Paktia
3	Herat	Balkh

**Table 2:** **Pilot and comparison districts**

No.	Intervention districts	Comparison districts
1	Narkh	Dimirdad
2	Jalrez	Beshood-1
3	Sayedabad	Chak
4	Ismailkhail-Mandozai	Tanni
5	Alisher-Terezay	Mosakhail
6	Qarabagh	Shakardara
7	Istalif	Guldara
8	Eshkamish	Khwaja Ghar
9	Garmser	Khanashin
10	Spin Boldak	Maiwand
11	Qaysar	Sherin Tagab

These three provincial and eleven district committees were consulted through four 2-day workshops. The consultations helped shape the draft guides focused on how to cultivate accountability, engage with diverse stakeholders, set a shared strategic direction, and steward resources to make the health system more responsive to the needs and expectations of the people. These effective governing practices were at the heart of the guides. The guides provided broad guidance on how to apply these practices in the PPHCC and DHCC governance in order to improve the performance and responsiveness of the provincial and district health systems. The MOPH team approved the contents of the governance guides for the purpose of testing.

### Second phase: participatory development of health systems governance development action plans and baseline measurement of governance

Three PPHCC and eleven DHCC teams participated in four 2-day workshops facilitated by public health and governance experts. The first day was spent working in groups discussing governance actions to apply the four effective governing practices in their work over the next six months to better meet the health needs and expectations of the people. Each committee using the governance guide and the framework of four effective governing practices identified actions to be taken in the next six months to improve their governance and developed its governance development action plan. The four practices provided the organizing framework to structure their committee deliberations and governance development action plans. On the second day, participants carried out a self-assessment of their governance performance at baseline. A pilot-testing protocol for the guide containing precise steps to be taken during the testing period was developed in consultation with the pilot PPHCCs and DHCCs. Some examples of actions planned by the committees are illustrated in Table 3.

**Table 3:** **Examples of activities selected by the PPHCCs and DHCCs to implement effective governing practices**

Governing practice	Examples of activities
Improve stakeholder engagement	**•** Interview patients and health service users
	**•** Invite religious, youth, and women leaders to meetings
	**•** Provide feedback to consultative assemblies at health facility level
	**•** Consult community leaders on a regular basis
Cultivate accountability	**•** Share information on resources and performance with communities and stakeholders
	**•** Encourage health workers to share their challenges during joint monitoring visits
	**•** Review health workers’ job descriptions and provide clear service delivery targets
Set a shared strategic direction	**•** Constitute a team of representatives from the community, health service users, other health system stakeholders, and district health officers from each district to identify the health needs and challenges faced by the communities, and to communicate these needs to the PPHCC for consideration while deciding on the strategic direction
	**•** Invite health facility shura members to meetings to better understand community health concerns
Steward resources responsibly	**•** Train provincial public health office staff and health workers in ethics
	**•** Recognize health workers with outstanding performance
	**•** Involve the community in health facility monitoring
	**•** Use data, information, evidence, and technology for decision making

We developed five self-assessment instruments to measure governance performance: two to be used by committees as a whole, one by the chair and two by individual members to assess their governance performance. We designed these instruments based on our conceptual model and the current role of the committees, and also the expanded role they aspired to take on to make their governance more effective and people-centred. The five measurement instruments are summarized in Table 4 and available in full in the Additional file 1.

**Table 4:** **PPHCC and DHCC governance measurement instruments used in the study**

#	Name of the governance self-assessment scale	Based on	Administered to whom	Frequency of administration	What is measured
1	Overall PPHCC/DHCC governance performance assessment scale	Roles and responsibilities of the PPHCC/DHCC	PPHCCs and DHCCs (committee as a whole did collective self-assessment)	Pre- and Post-intervention	PPHCC and DHCC governance
2	Governance standards-based scale	Governance standards	PPHCCs (committee as a whole did collective self-assessment)	Pre- and Post-intervention	PPHCC governance
3	PPHCC/DHCC Chair’s self-assessment scale	Roles and responsibilities of the chairperson	PPHCC and DHCC chairpersons	Pre- and Post-intervention	Chairperson’s governance performance
4	Individual member practice-based 30-item scale	Governing practices	Individual members of the PPHCCs and DHCCs	Pre- and Post-intervention	Governing practices of the PPHCC and DHCC members
5	Individual member competency-based scale	Governance competencies	Individual members of the PPHCCs and DHCCs	Pre- and Post-intervention	Governance competencies of the PPHCC and DHCC members

PPHCCs used two self-assessment scales for assessing governance of the committee as a whole. One of them was the overall health governance instrument for self-assessment of performance on their governance responsibilities. The three PPHCCs graded their own performance on a 1–10 scale on each responsibility of the committee. The other instrument assessed PPHCC health governance standards based on 11 provincial public health core functions. No progress on a standard was scored 0, 1-25% accomplishment was scored 1, 26-50% was scored 2, 51-75% was scored 3, and 76-100% was scored 4. Individual members used two self-assessment instruments, one based on the four practices of effective governance, and the other based on their governing competencies. Chairpersons of the committees self-assessed how well they were carrying out their responsibilities as chairs using a separate instrument.

DHCCs used similar measurement instruments except they did not have a health governance standards-based scale because a core functions framework for district health offices does not exist.

### Third phase: implementation and monitoring

In implementing their governance development action plans, the three PPHCCs and eleven DHCCs worked to improve engagement with the public and communities, and to become more transparent, accountable, and responsive. No additional resources were made available to the provinces and districts to carry out their planned activities. Committees monitored implementation of their action plans so underperformance could be identified and corrected along the way. The actions in the plan were monitored on a monthly basis using a simple Excel-based monitoring instrument. Progress report was sent to the Provincial Liaison Directorate of the MOPH.

The PPHCCs and DHCCs monitored their progress by the extent to which actions were implemented. Progress on an action or activity was classified in five categories: Not started (0%), early stage (1-25% of an action is completed), two intermediate stages (26-50% or 51-75% of an action is completed), and advanced stage of completion (76-100% of an action is completed).

### Fourth phase: evaluation

The PPHCCs and DHCCs in the pilot evaluated their performance during the pilot-testing period in four 2-day workshops held six months after the testing began i.e. at the conclusion of the pilot. PPHCC and DHCC re-assessed their governance performance as a committee and as individual members using the same instruments they had used at baseline before the pilot test began. Eleven focus group discussions were held with the three provincial and eleven district health coordination committee members to explore their successes and challenges during the pilot testing period, to discuss the applicability of the pilot approach based on the four effective governing practices to their situation, and to make specific recommendations to the MOPH.

Select health system performance indicator data were collected for the pilot and comparison districts and provinces to examine the impact on health systems performance. We collected HMIS data on seven indicators of health systems performance (proportion of pregnant women who received two doses of tetanus toxoid, proportion of facility deliveries, proportion of pregnant women who received at least one antenatal care visit, proportion of new mothers who received at least one postnatal care visit, TB case detection rate, community health worker home visit rate, and proportion of new family planning users in target population), and one health outcome (TB cure rate), since TB is highly prevalent in the region and is a public health priority.

We expected to see change in maternal and child health indicators because maternal and newborn care, and child health and immunization are the top two of the seven elements of the Basic Package of Health Services, and this package is the mainstay of the primary health care in Afghanistan. Any systemic improvements in the provincial and district health systems are expected to reflect in the indicators related to maternal and child health.

We used difference-in-differences strategy to draw an inference. In difference-in-differences methodology, outcomes are observed for two groups for two time periods, pre and post. One group is exposed to an intervention while the other is not. The difference in the control group is subtracted from the difference in the intervention group. This methodology removes biases in the post period comparisons between the intervention and control group that could result from permanent differences between the groups, as well as biases from comparisons over time in the intervention group that could be the result of a time trend [26].

We collected security updates from a NGO safety organization to keep track of security situation in the intervention provinces and districts.

#### Measurement challenges

A disadvantage of self-assessment as a method of obtaining data is a greater chance of measurement error [27]. In a meta-analysis of 44 self-assessment studies in higher education, Falchikov and Boud reported correlations between self-assessed and external measures of performance ranging from -0.05 to 0.82, with a mean correlation of 0.39 [28]. In a similar review of 18 self-assessment studies in the health professions, Gordon reported correlations ranging from 0.02 and 0.65 [29]. In both sectors, the coefficients of correlation had a very wide range i.e. correlation was nominal to very high. This can happen because of unintentional and intentional measurement errors. Unintentional errors arise when questions are unclear or ambiguous, when there are limitations to respondents’ comprehension or memory, or when the measurement scales used are not clear. Respondents might intentionally alter their true responses because of social desirability, boastfulness, or modesty [30].

We made two amends. One, we acknowledge the existence of bias in measurement by clearly reporting limitations of data. Two, we mitigate this bias by avoiding items composite of several underlying dimensions, and by defining assessment questions as clearly as possible. Going beyond to further reduce bias, we could have used a combination of different methods.

Gathering the perspectives of stakeholders who were not part of the PPHCC or DHCC could have provided subjective assessment data from external sources. Collecting data on objective indicators that measure governance process in some way (for example, whether a governing body met every month, attendance at governing body meetings, number of decisions taken, etc.) could have provided quantitative data, not necessarily spanning all the dimensions of governance, to complement our data.

Governance self-assessment scores across multiple dimensions of governance were the best measures we had for assessing governance. There are no gold standard measures of governance that have already been tested for reliability and validity. This remains an area for future governance research.

We use health system performance indicators of health service access and utilization that measure, at least in part, the effectiveness of the health system in reaching its beneficiaries. Efficiency in the use of resources could be measured if cost data were available. Cost per output and cost per outcome data are not readily available in Afghanistan. Collecting these data would have required additional resources that we did not have. We did not measure social and financial risk protection, responsiveness of the health system, or efficiency of the health system as organizational outcomes of better governance because this would involve conducting resource-heavy representative surveys. Health system performance indicators by wealth quintile and sex-disaggregated data are not readily available in the HMIS, and costs of primary data collection are very high, so these measures were also not included in this assessment.

## Results

### Governance development action plan implementation

The PPHCCs and DHCCs implemented many specific governance actions to better meet the health needs of the people. At the end of the six month pilot testing period, they assessed their performance of these actions (Table 5).The committees also discussed whether they plan on continuing each action beyond the pilot testing period. We defined an action or activity sustainable in the short term if the committee decided to continue implementing it beyond the duration of the pilot.

**Table 5:** **Progress on implementation of governance development action plans at the end of six months of pilot-testing**

Governing practice	No. of actions	Percent completion					Short-term sustainability* (no. of actions)	Short-term sustainability (%)
		0%	1-25%	26-50%	51-75%	76-100%		
		Not started	Early stage	Intermediate stage		Near- complete or complete		
**Wardak, Khost and Herat PPHCCs**								
Engaging stakeholders	158	16	9	19	23	91	151	96%
Cultivating accountability	65	0	1	8	11	45	63	97%
Setting a shared direction	56	1	1	2	14	38	53	95%
Stewarding resources	93	7	10	8	25	43	91	98%
*Total number of specific actions*	372	24	21	37	73	217	358	96%
*Percentage of actions at various stages*		6.5%	5.6%	9.9%	19.6%	58.3%		
**Eleven DHCCs**								
Engaging stakeholders	588	98	58	71	97	264	488	83%
Cultivating accountability	274	7	30	44	63	130	246	90%
Setting a shared direction	127	7	5	25	38	52	97	76%
Stewarding resources	218	35	18	34	36	95	201	92%
*Total number of specific actions*	1207	147	111	174	234	541	1032	86%
*Percentage of actions at various stages*		12.2%	9.2%	14.4%	19.4%	44.8%		

*Legend*

*Short-term sustainability of an action indicates that the respective committee decided at the time of end-of-pilot measurement to continue it in future even after the end of pilot testing phase.

**Figure 2:**
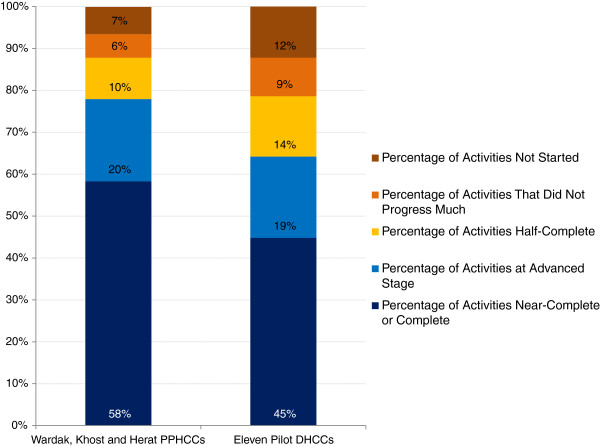
**Progress on implementation of governance development action plans at the end of six months of pilot testing.** *Legend*: The PPHCCs accomplished 58% of their governance development action plan, and the DHCCs completed 45% of their action plan.

Table 5 and Figure 2 show that the pilot PPHCCs and DHCCs reported a high level of completion of their governance development action plans. In six months, three provinces on average accomplished 58% of their action plan and in addition 30% was at an intermediate stage. The eleven districts on average accomplished 45% of their action plan and in addition, 34% was at an intermediate stage of completion. The actions had a high level of short-term sustainability; committees plan on continuing 76-98% of these actions in the future.

### Governance measurement

**Figure 3:**
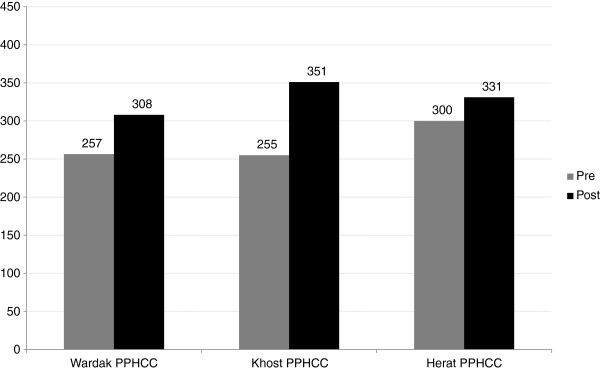
**PPHCC governance self-assessment scores.** *Legend:* 1. PPHCCs improved their governance score on average by 13.2%. 2. Khost PPHCC improved their governance score by 21%, Wardak by 11% and Herat by 7%. 3. Scale used: PPHCC overall governance performance scale. 4. Total governance score possible: 450.

**Figure 4:**
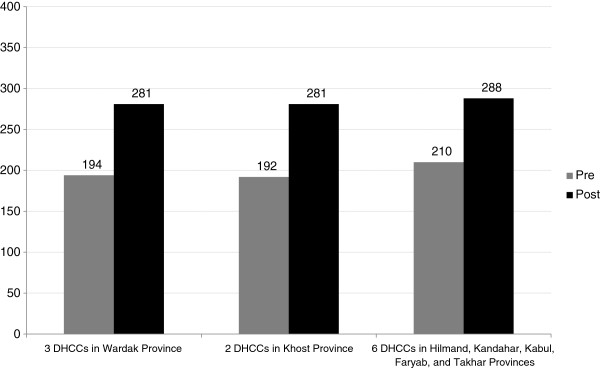
**DHCC governance self-assessment scores.** *Legend:* 1. DHCCs improved their governance score on average by 20.6%. 2. Scale used: DHCC overall governance performance scale. 3. Total governance score possible: 400.

PPHCCs (see Table 6 and Figure 3) and DHCCs (see Table 7 and Figure 4) self-assessed their governance performance at baseline and again after six months of pilot testing. Overall, we found significant improvements in governance scores. PPHCCs improved their governance score on average by 13.2% and 18.5% using two different scales (one based on roles and responsibilities and the other on governance standards), and individual PPHCC members improved their governance score on average by 6.7% and 9.7%, also using two different scales (one based on governing practices and the other on governance competencies). DHCCs improved their governance by more than 20%.

**Table 6:** **PPHCC governance self-assessment scores**

#	Governance self-assessment scale	Maximum score	Wardak			Khost			Herat			Overall percent point change
			Pre	Post	Percent point change	Pre	Post	Percent point change	Pre	Post	Percent point change	
1	PPHCC overall governance performance scale	480	256.5	308	11.44	255	351	21.33	300	331	6.89	13.2
2	Governance standards-based scale for the PPHCC	184	91.5	132	22.01	96.5	143	25.27	109	124	8.15	18.5
3	Chair	80	43	72	36.25	49	76	33.75	70	73	3.75	24.6
4	Individual member practice-based 30-item scale*	280	229	233	1.43	215	265.5	18.04	244	248	1.43	7.0
5	Individual member competency-based scale*	72	53	55	2.78	46	61	20.83	57	61	5.56	9.7

*Legend*

*Average of individual scores of all members of a committee.

**Table 7:** **DHCC governance self-assessment scores**

#	Governance self-assessment scale	Maximum score	Wardak province			Khost province			Other provinces			Overall percent point change (Weighted average)
			Average of three districts			Average of two districts			Average of six districts			
			Pre	Post	Percent point change	Pre	Post	Percent point change	Pre	Post	Percent point change	
1	DHCC overall governance performance scale	400	194	281	21.75	192	281	22.25	210	288	19.50	20.6
2	Chair	80	0	64	80	41	56	18.75	56	63	8.75	30.0
3	Individual member practice-based 30-item scale*	280	144	262	42.14	213	262	17.50	226	259	11.79	21.1
4	Individual member competency-based scale*	72	17	60	59.72	43	45.8	3.89	47	53	8.33	21.5

*Legend*

*Average of individual scores of all members of a committee.

Improvement was higher in the provinces and districts where leaders were more committed to a people-centred approach as inferred from the extent of completion of their governance development action plans. Khost PPHCC improved their governance score by 21%, Wardak by 11% and Herat by 7%. We found that action plan completion (completion above 50%) was in the same rank and order (Khost 89%, Wardak 83%, and Herat 66%).

### Health system performance

On analyzing data for seven indicators of health system performance and one health outcome indicator in pilot and comparison provinces and districts (see Table 8), we did not find a statistically significant impact of the intervention on health system performance or health outcomes, except antenatal care visit rate in the pilot provinces increased by 20%. Many indicators worsened in the intervention group. However, these changes were not statistically significant (p ≥ 0.15).

**Table 8:** **Health system performance and health outcomes**

#	Performance indicator (Rates)	Intervention provinces				Comparison provinces				Difference-in-differences
		Pre	Post	Difference	p-value*	Pre	Post	Difference	p-value*	
**Health system performance**										
1	TT2+ rate	275	176	-99	0.10	280	177	-103	0.15	4
2	Facility delivery rate	11	14	3	0.47	21	27	6	0.32	-3
3	One ANC visit rate	54	126	72	0.05	87	139	52	0.03	20
4	One PNC visit rate	95	75	-20	0.29	118	106	-12	0.37	-9
5	TB case detection rate x 100	105	97	-8	0.86	89	84	-6	0.87	-3
6	CHW home visit rate	29	30	1	0.96	43	43	0	0.97	1
7	New FP rate	17	18	0	0.96	19	22	4	0.71	-3
**Health outcome**										
8	TB Cure Rate	103	86	-16	0.31	87	86	-1	0.92	-15
	**Performance indicator (Rates)**	**Intervention districts**				**Comparison districts**			p-value*	**Difference-in-differences**
**Health system performance**										
1	TT2+ rate	74	89	15	0.63	59	83	24	0.28	-9
2	Facility delivery rate	12	12	0	0.87	16	19	3	0.65	-3
3	One ANC visit rate	103	103	1	0.92	88	101	13	0.49	-13
4	One PNC visit rate	64	73	9	0.82	55	53	-2	0.89	10
5	TB case detection rate x 100	59	79	19	0.68	44	79	35	0.15	-16
6	CHW home visit rate	29	30	1	0.81	34	33	-1	0.88	2
7	New FP rate	12	12	0	0.40	12	11	-1	0.65	1
**Health outcome**										
8	TB Cure Rate	123	149	26	0.63	34	50	17	0.45	9

*Legend*

1. TT2+ rate is percentage of pregnant women who have received TT2+ vaccine.

2. Facility delivery rate is percentage of pregnant women delivered at the health facility.

3. One ANC visit rate is percentage of pregnant women who have received at least one antenatal care visit.

4. One PNC visit rate is percentage of new mothers who have received at least one postnatal care visit.

5. TB case detection rate is Tuberculosis case detection rate.

6. CHW home visit rate is calculated as number of home visits × 100/Number of Target visits in a month.

7. New FP rate is New Family Planning users calculated as number of New FP Users in the month × 100/Monthly Target Population.

8. TB Cure Rate is calculated as number of cases that completed treatment and were smear negative divided by the number that started treatment × 100.

*p-value obtained on conducting two-sample equal variance two-tailed *t*-test.

### Focus group discussions

We held eleven focus group discussions with the provincial and district health coordination committee members at the end of the pilot testing period. The committees reported many achievements and successes. These achievements are in several key areas clustered around the effective governing practices.

1. *Enhanced transparency and accountability*: PPHCC and DHCC members noted that their meetings had become more regular; had clear agendas; and minutes of meetings were documented and made public. The members reported that new sub-committees that oversee accountability, for example financial audit and transparency subcommittee and governance subcommittee were established at the province level; information and decisions of the committees were shared through emails, press conferences, magazines, newsletters and social media websites; private sector pharmacies and food stores that lacked legal documents or did not follow regulatory standards were closed down; expired medicines were rounded up through monitoring visits; and judicial proceedings were started against corrupt health facility personnel.

The members stated that their activities as a committee became more transparent and involved diverse stakeholders; intersectoral coordination and collaboration improved; there was an improved focus on patients’ health problems at the community level; community health needs were defined, and discussed; coordination, communication, and information sharing improved; the frequency of data analysis and presentation to the committees increased, and more committee decisions were evidence based.

2. *Inclusion of stakeholders and stakeholder views*: The PPHCC and DHCC members reported that steps were taken to recruit more women to community health nursing educator posts; suggestion and complaint boxes were placed outside health posts and the community complaints were discussed during regular meetings; attendance of members at the meetings improved; civil society groups, community leaders and representatives from other sectoral departments were invited to the committee meetings; community concerns were discussed as a standing agenda item during committee meetings; and vaccination rejection in some villages was addressed by negotiating with elders and through community mobilization efforts.
3. *Setting shared strategic direction*: The PPHCC and DHCC members reported that the MOPH guidelines and policies were introduced and shared in the PPHCC and DHCC meetings; service delivery data was analyzed and shared more frequently with committee members; and annual plans were developed and shared with the stakeholders in the province and the MOPH.
4. *Stewardship of resources*: A committee reported success in mobilizing resources to build toilets in the health facility and completing the overdue construction of a community health center. Health center buildings and facilities were improved through community support at a few places, and inspections and enforcement of quality standards also improved.

The members observed that there was a better link between committees at provincial and district levels. Communities began participating in health facility monitoring, gave feedback on the quality of health services, and became motivated to contribute to health service delivery improvements.

There were also goals that committees wanted to accomplish but could not, for example better coordination between shuras or committees at different levels and more effective communication with the public. Some of the actions in the governance development action plan needed substantial extra resources which the committees did not have. Overall, lack of resources emerged as one of the most common challenges the committees faced in completing their action plans. The security situation often prevented engagement of provincial and district governors, and also did not allow for the level of health facility monitoring that the committees would have liked to achieve. Resources for training and education of committee members were found to be grossly inadequate. Community expectations on health service delivery rose, and the committees did their best to meet these expectations by mobilizing community support and resources.

Overall, committees reported notable changes in their knowledge, skills, and behaviors, including feeling more capable, responsive, and accountable than they were before the intervention. Committee functioning became more systematic and regular, and members felt more responsible for their decisions. Committees also noticed improvements in their effectiveness; referral of TB cases for treatment improved in one district, un-served remote areas were identified, and 90% of them were covered through establishment of mobile teams in another district. One committee reported that antenatal and postnatal care visits increased, and other maternal and child health services improved. Because of increased community engagement, committees felt they could solve problems at the health facility level in collaboration with the local community. This experience showed them that they could build trust with the communities by working with them.

Committee members said they would continue applying effective governing practices in the future mainly because they felt their achievements in the short six month period were encouraging. They became aware of weaknesses in their governing and resolved to improve. Members thought they gained many benefits at the individual level because of changes in their attitudes and behavior. They also became aware of their stewardship role and want to do more for the communities they serve. The intervention, PPHCC and DHCC members believed, renewed their commitment to their governance responsibilities. They observed that periodic governance assessments and the overall pilot experience developed their capacity in discharging their governance role. The committees recommended that the MOPH should officially introduce the piloted approach in all the provinces and districts, and expressed interest in sharing their governance development experience with other provinces and districts.

### Security challenges

Implementing governance interventions in the fragile and conflict affected environments presents a significant challenge. Opposition groups are hostile to anyone linked with the government. During the pilot testing phase, the security situation remained unpredictable and volatile and the pilot provinces and districts witnessed considerable activity by armed opposition groups. In Nerkh district, a rocket struck the compound of a clinic and broke the window panes during an armed clash between opposition groups. Five opposition operatives wearing body-borne explosive devices mounted an armed attack and detonated a truck-borne improvised explosive device (IED) in the vicinity of the provincial public health office and provincial hospital in Wardak province. Staff and patients were injured by broken glass, and the chairperson of the Wardak PPHCC and a committee member suffered minor head injuries. Security challenges in this case directly affected PPHCC leaders. In Jalrez district, two rental trucks transporting medical supplies from Kabul to health clinics were hijacked by an armed opposition group, and one truck with medical supplies was later released through mediation and support from local elders. Opposition groups and organized criminal elements abducted many NGO health staff, and there were several armed clashes and armed attacks in the districts of Herat province. Qaysar district experienced armed clashes and attacks and an IED explosion in the vicinity of a clinic. The modest gains achieved in governance during this pilot-test become especially noteworthy in view of this tough security environment.

## Discussion and evaluation

We piloted an intervention that placed a health systems governance approach in the hands of multi-stakeholder committees that govern provincial and district health systems, using the organizing framework focused on four effective governing practices. We sought to explore whether and how health systems governance can be improved in fragile and conflict affected environments, and found that it can be. We measured the self-reported PPHCC and DHCC governance scores before and after the intervention, and found strong evidence that their governance scores and practices improved following the intervention. PPHCC governance score improved by 13 percentage points, and DHCC governance score improved by 21 percentage points. The commitment of the PPHCCs and DHCCs to continue implementing, at least in the short term, 96% and 86% of their governance improvement plans, respectively, is encouraging. The intervention was associated with a 20% increase in the antenatal care visit rate in the pilot provinces. We did not find any other quantitative evidence of improved health system governance leading to higher health system performance, we believe because the intervention was of a short duration.

### Lessons learned

#### Framework of effective governing practices makes governance enhancement accessible to the leaders

Leaders who govern may want to improve their governance but may not know how to do it. In our intervention, the four governing practices - *cultivating accountability, engaging with stakeholders, setting a shared strategic direction, and stewarding resources* - provided an organizing framework to the governance leaders to plan their governance enhancement. When the provincial and district health coordination committee members, who have a predominant governing role, designed and implemented their governance development action plan based on the four practices, their governing behavior and consequently the governance of their provincial and district health systems improved. The intersectoral and inter-departmental collaboration received a boost because of better stakeholder engagement; this is highly relevant in the context of health as the work of many ministries and sectors other than health influences health status of the populations.

#### Participation of the governance leaders enhances their commitment

The provincial and district health coordination committee members designed the intervention in a participatory and consultative manner. This created a sense of responsibility in them to implement during the intervention period the governance development action plan they had created at the beginning of the intervention. When the leaders who govern make their own plan of governance improvement, they are more likely to be committed to implement it.

#### Placing people at the center of the intervention brought life to it

People who govern, health managers, health providers, health workers, community leaders, and patients were at the center of the intervention. The intervention was focused on health system leaders governing in close partnership with health managers, health providers, health workers, community leaders, patients, and governance leaders in other sectors. The intervention was organized based on the health needs and expectations of people and communities, rather than by vertical disease programs. This helped make the intervention meaningful for the governance leaders.

#### Governing bodies at decentralized levels can represent community concerns and resolve them

Centralized health systems, by their structure and organization, make it challenging for people at the sub-national level to engage with the health system and influence it so that the health services that people and communities need are available and accessible to them. As health systems become decentralized, sub-national structures and committees are entrusted with a responsibility to coordinate, monitor, and oversee health services; they are expected to play a governance role and have an opportunity to make their governance, and in turn their health systems, people-centred since they are closer to the people. Governing bodies at community level can represent unresolved community health needs to governing bodies at district and provincial levels which can address them in time.

#### Governance improvements need time to translate into improved health system performance

Governance intervention is feasible in fragile and conflict affected environments and should be implemented over sustained periods of time in order to realize gains in the health system performance. The effective governing practices need to be consistently applied, periodically assessed and continuously improved. Governance improvements need time to translate into improvements in health system performance at health facility level. Duration of our intervention was too short to impact health system performance or health indicators.

#### Leadership of the ministry matters

The MOPH faces significant challenges. The ministry was short of staff and capacity. It did not have enough staff to support monitoring of the governance intervention. Nor did it have much experience in improving its own governance. Some actions selected by the PPHCCs and DHCCs needed extra budget which the ministry could not provide because of resource constraints. Armed conflict affected health providers’ and workers’ safety and ability to provide quality health services at facility and community levels for which the ministry could not do much. District Health Offices and DHCCs were less well established compared to the Provincial Public Health Directorates and PPHCCs; the ministry did not have adequate resources to equip them well. Despite these challenges, the leadership and involvement of the ministry in the intervention mattered. The provincial and district health governance leaders were inspired to improve their governance because the ministry leaders were interested in the pilot intervention.

### Limitations

Our study has limitations. Firstly, we were able to establish the face validity and content validity of the governance self-assessment instruments we used through reviews by subject matter experts. But we could not test their reliability. Examining reliability and validity of the instruments is a potential area of future research. Secondly, there is an element of subjectivity in the self-assessments. Individual self-assessments are more vulnerable to subjectivity than group self-assessments because group process can moderate over-rating; if one member of a group over-rates performance on an item, another group member can bring this to the attention of the group which can in turn affect the group’s final rating of that item. Thirdly, community members were not participants in our focus groups, so the community perspective was not reflected in the discussions. PPHCCs and DHCCs had engaged with health facility councils and community health councils in their areas, but the perspectives of these councils at community and facility levels is not reflected in the study as we could not survey or interview them, mainly because of geographical distances and high levels of insecurity in many of the rural areas. Fourthly, Hawthorne effect, i.e. governance improved because governance leaders modified their behavior for the purpose of the assessment rather than as a result of the intervention, could be a plausible alternative explanation for the results. Governance self-assessments in comparison jurisdictions could have either refuted or established the Hawthorne effect as the reason for improvement in governance scores. We did not carry out these assessments. Finally, our intervention was based on technical dimensions of governance and did not consider its political dimensions, for example, questions of political will, political power relationships, etc.

Our study also has several strong points. It contributes a conceptual model of health systems governance based on four effective governing practices in the context of low and middle income, especially fragile and conflict-affected countries. We have documented a strategy to promote people-centred health systems governance in resource-constrained and insecure environments and generated empirical evidence in this respect. We detailed a participatory approach to health systems governance where health system leaders identified and acted on opportunities for strengthening their health systems, and making themselves and their health systems more accountable and responsive.

## Conclusion

Our findings have implications for policy and practice within and beyond Afghanistan. We found that health systems governance can be improved even in fragile and conflict affected environments. We infer that governance is central to making health systems responsive to the needs of people who access and provide health services. When health governing bodies in the communities, district, and province work in coordination, community health concerns can be effectively represented and addressed and health system can become more responsive to the community needs within the available limited resources. The approach based on the four effective governing practices helps the provincial and district health coordination committees perform their governance roles and responsibilities in a more effective, efficient, transparent, and accountable manner. This has a potential to influence not only patient care experiences in the provinces and districts, but also access to care, quality of care, and overall health outcomes for people and communities.

The pilot PPHCCs and DHCCs aspire to continue applying the effective governing practices, and have recommended that the MOPH scale up the approach nationally. The MOPH Provincial Liaison Directorate is supportive of this recommendation and has recently introduced this approach in 13 more provinces with the MOPH concurrence. Provinces and districts will need support in the initial stage in terms of assistance in orientation and governance enhancement action planning. The MOPH should actively support the approach. Provincial Public Health Director and District Health Officer work plans should include governance enhancement, and their performance should be assessed every year on this role, along with other public health responsibilities. The donors of the MOPH also should support the health systems governance enhancement work in the MOPH as well as in the provinces and districts. Leadership, management and governance education should be included in the curriculum of the medical, nursing, and public health disciplines, and in the in-service training of the MOPH employees.

Our study provides a potentially useful approach to improving governance of health systems in a fragile and conflict affected environment. These study results may be applicable to similar settings where governing bodies or committees governing provincial or district health systems wish to adopt people-centred health systems governance approaches. Future studies should consider longer duration and more rigorous research design that test whether improved health systems governance leads to higher health system performance and improved health outcomes.

## Electronic supplementary material

Additional file 1: **Five Measurement Instruments Used in the Study.** (PDF 791 KB)

## Abbreviations

PPHCC: Provincial Public Health Coordination Committee
DHCC: District Health Coordination Committee
MOPH: Ministry of Public Health
NGO: Nongovernmental Organizations
UNICEF: United Nations Children’s Fund
WHO: World Health Organization
HMIS: Health Management Information System
TB: Tuberculosis
TT2+: Second and subsequent doses of tetanus toxoid
ANC: Antenatal care
PNC: Postnatal care
CHW: Community health worker
FP: Family planning.
