# Supplementary material for: Implementing people-centred health systems governance in 3 provinces and 11 districts of Afghanistan: a case study
Source: Confl Health. 2015 Jan 7;9:2. doi: 10.1186/1752-1505-9-2 (PMC4406217; doi:10.1186/1752-1505-9-2)
Supplement: Supplementary file 1 — Additional file 1: Five Measurement Instruments Used in the Study. (PDF 791 KB) [file 13031_2014_147_MOESM1_ESM.pdf]

Additional file 1

1. Overall Health Governance Self-Assessment Tool for the PPHCC

Directions: For each item, note briefly the internal strengths and weaknesses within the PPHCC as well as the opportunities and threats that exist in the current environment. You will rate performance on a scale of from 1 to 10, with 10 being the best possible rating.

#	Responsibility	Internal		External		Performance (scale 1–10)
		Strengths	Weaknesses	Opportunities	Threats	
**Role and responsibilities as per ToR**						
1	Meeting the MOPH policies, priorities, objectives, strategies, and standards: a. Overall					
	b. In relation to the BPHS					
	c. In relation to the EPHS					
2	Coordination of all stakeholders: a. At the provincial level					
	b. In the districts and the communities					
	c. With the MOPH					
3	Improvement of services: a. Overall quality					
	b. Accessibility					
	c. Sustainability					
4	Input into provincial planning, consolidation of the provincial plan, and monitoring of its implementation					
5	Oversight: Development and implementation of a joint supervision plan					
6	Coordinate an emergency response and participate in it					
7	Coordinate and participate in immunization campaign					
8	Provide technical and consultation support to the PPHD					
9	Sharing of information related to service delivery, MOPH policies, strategies, standards, new initiatives, and any problems and challenges					
10	Identify sites for new health facilities					
11	Mediate disputes that arise among stakeholders and ensure shared understand-ing between governmental organizations and NGOs (cases referred by DPHO)					
12	Follow up of the work of committees: a. HMIS Committee					
	b. EPI Task Force					
	c. Emergency Task Force					
	d. CDC Committee					
	e. Reproductive Health Committee					
	f. Maternal and Child Survival Committee					
	g. Child and Adolescent Health Committee					
	h. Nutrition Committee					
	i. Pharmacy Committee					
	j. Environmental Health Committee					
	k. Any other provincial health-related committee					

**Expanded role and responsibilities**						
13	Advocate and ensure openness, transparency, accountability, honesty, and inclusiveness:<br>a. In the health system of the province					
	b. In the affairs of the provincial health directorate					
14	Set strategic direction:<br>a. For 3–5 years					
	b. Strategic plan is based on the strategic direction					
15	Advocate and support achievement of health outcomes, responsiveness and patient satisfaction, and patient safety					
16	Nurture relationships with the communities and the people served					
17	Stewardship of scarce resources:<br>a. Ethical and best use of available resources for achievement of health outcomes for the people served					
	b. Exert influence across different sectors for achieving best health outcomes for the population in province					
	c. Provide vision and direction for health systems					
	d. Collect and use information and evidence on health system performance to ensure accountability and transparency					
18	Ensure adequate financial resources					
19	Provide financial oversight					
20	Monitor performance					
21	Support high performance					
22	Strengthen health services in the province					
23	Continuous improvement of the functioning of the PPHCC					
24	Build a competent PPHCC					
25	Advocate for decentralization					
26	Any other responsibility PPHCC fulfills					

Scoring criteria

The maximum score that can be earned is 450.

- Score of 338 and above: Outstanding governance
- Score of 226–337: Meets most requirements
- Score of 113–225: Needs improvement
- Score below 113: Unsatisfactory governance

2. Health Governance Standards-based Self-Assessment Tool for the PPHCC

This instrument is based on 11 PPHO core functions and 46 PPHC governance standards.

Scoring guide

0%	1%–25%	26%–50%	51%–75%	76%–100%
**No activity**	**Minimal activity**	**Moderate activity**	**Significant activity**	**Optimal activity**
0%, or absolutely no activity	Greater than zero, but no more than 25% of the activity described within the standard is met	Greater than 25%, but no more than 50% of the activity described within the standard is met	Greater than 50%, but no more than 75% of the activity described within the standard is met	Greater than 75% of the activity described within the standard is met

Instrument

#	ToR	0%	1%–25%	26%–50%	51%–75%	76%–100%
**I**	**Oversight for health situation and trend assessment**					
1	Facilitates access to appropriate resources for community health status monitoring, and mobilizes resources and support for the surveys that are centrally designed					
2	Promotes broad-based participation and coordination among all entities active in collecting, analyzing, and disseminating community health status data					
3	Provides oversight and support for community health status monitoring efforts					
4	Guides improvements in the health status monitoring efforts					
**II**	**Oversight of monitoring and evaluation of health services**					
1	Facilitates access to the necessary resources to conduct periodic monitoring and evaluations					
2	The PPHCC itself evaluates the health services					
3	Makes sure that regular supportive supervision, monitoring and evaluation of health services provided in the public and the private sectors in the province take place					
4	Encourages all relevant stakeholders provide input into monitoring and evaluation processes					
5	Reviews evaluation results and utilizes these results to improve health service performance					
**III**	**Oversight for data and information management**					
1	Facilitates access to appropriate resources for data and information management					
2	Promotes broad-based participation and coordination among all entities active in data and information management tasks					
3	Reviews data and information management on a quarterly basis, and provides oversight and support for data and information management efforts					
4	Guides improvements in overall data and information management					

#	ToR	0%	1%–25%	26%–50%	51%–75%	76%–100%
**IV**	**Oversight for health service delivery**					
1	Oversees public and nongovernmental organizations and the private sector responsible for delivery of health services					
2	Allows community monitoring of the delivery of health services					
3	Facilitates community input in problem identification and problem solving					
4	Conducts periodic reviews of health service delivery with special attention to services for vulnerable populations					
**V**	**Oversight of coordination, communication, and intersectoral collaboration activities**					
1	Facilitates access to national, state, and local resources that could be used in support of these activities					
2	Establishes and oversees the implementation of policies to support activities to inform, educate, and empower people about public health issues, and reviews these activities in light of community needs					
3	Makes sure that all population subgroups have an opportunity to provide input on health issues and health services					
4	Exerts influence across sectors to protect and promote health of the community					
**VI**	**Supporting health service delivery**					
1	Ensures that the PPHO is supporting the implementing NGOs through joint visits, assessment of quality of service, and assistance in staff recruitment and training					
2	Encourages members from lead and secondary NGOs to surface their issues in a timely fashion					
3	Mobilizes community support of the NGOs delivering appropriate services					
4	Recognizes NGOs delivering quality services to vulnerable populations					
**VII**	**Oversight of health resource management**					
1	Establishes and oversees the implementation of policies designed to assure efficient and effective use of physical, financial, and human resources and drugs and supplies					
2	Establishes and oversees the implementation of policies designed to assure improvements in workforce, management, and leadership quality					
3	Facilitates access to national, state, and local resources available for workforce training, leadership development, and continuing education					
4	Provides for the training and continuing education of the PPHCC					
5	Assists in mobilizing resources for the provincial health system and the public health services					
**VIII**	**Oversight of preventive and clinical services, environmental health services, and forensic medicine services**					
1	Oversees and supports the delivery of preventive and clinical services, environmental health services, and forensic medicine services					
2	Facilitates the community monitoring of the delivery of these services					

#	ToR	0%	1%–25%	26%–50%	51%–75%	76%–100%
3	Encourages community input into the delivery of these services					
4	Ensures transparency, accountability, and ethical and moral integrity in the provision of these services					
**IX**	**Oversight of strategic and annual planning**					
1	Maintains and annually reviews documentation of its mission statement					
2	Assesses and advocates for adequate resources and the MOPH’s support					
3	Supports planning for improvement in the health of the population in the province and works to strategically align community resources for this purpose					
4	Oversees implementation of the annual plan					
**X**	**Oversight of management of health emergencies**					
1	Supports planning for emergency response and works to strategically align community resources for this purpose					
2	Facilitates access to appropriate resources for management of health emergencies					
3	Promotes broad-based participation and coordination among all entities active in management of health emergencies					
4	Provides oversight and support for management of health emergencies					
**XI**	**Nurturing community relationship and involvement**					
1	Ascertains people’s preferences, needs, problems, challenges, and issues in health service delivery					
2	Mobilizes community input in the planning and implementation of the health services					
3	Mobilizes community input in monitoring, evaluation and ensuring accountability in health service delivery					
4	Provides relevant feedback to its stakeholders and the communities in the province					

3. **Practices of Health Governance Self-Assessment Tool**

This self-assessment tool is intended for use by individual members and is based on practices of effective governance.

Instructions: Please circle the choice that represents the extent to which you agree that the statement accurately describes your governance decision-making.

1. I do what I publicly or privately say.
Never 0 1 2 3 4 5 6 7 8 9 10 Always

1. I abuse my power for my private gain.
Never 0 1 2 3 4 5 6 7 8 9 10 Always

2. While making a decision in the PPHCC, I decide in favor of what I think is ethically right.
Never 0 1 2 3 4 5 6 7 8 9 10 Always

3. While making a decision in the PPHCC, I keep the interests of people in mind.
Never 0 1 2 3 4 5 6 7 8 9 10 Always

4. I scan the internal and external environment for strengths, weaknesses, opportunities, and threats to PPHCC priorities.
Never 0 1 2 3 4 5 6 7 8 9 10 Always

5. I focus my work and the work of those I lead on achieving the priorities of my PPHCC.
Never 0 1 2 3 4 5 6 7 8 9 10 Always

6. I mobilize human, material, and financial resources and align them to support priorities of my PPHCC.
Never 0 1 2 3 4 5 6 7 8 9 10 Always

7. I inspire those I lead to be committed to the PPHCC priorities.
Never 0 1 2 3 4 5 6 7 8 9 10 Always

8. I inspire those I lead to continuously learn how to adapt and do things better.
Never 0 1 2 3 4 5 6 7 8 9 10 Always

9. I believe in openness and transparency in the PPHCC decision-making process.
Not at all 01 2 3 4 5 6 7 8 9 10 Strongly

10. I believe in making information related to PPHCC decisions available to those who are affected by PPHCC decisions.

Never 0 1 2 3 4 5 6 7 8 9 10 Always

11. I believe in including those who are affected by PPHCC decision or their representatives in the PPHCC decision-making process.

Not at all 0 1 2 3 4 5 6 7 8 9 10 Strongly

12. I include those who are affected by PPHCC decisions or their representatives in the PPHCC decision-making process.

Never 0 1 2 3 4 5 6 7 8 9 10 Always

13. I am responsive to the different needs of men and women when I make a decision in the PPHCC.

Never 0 1 2 3 4 5 6 7 8 9 10 Always

14. I believe in the principle that PPHCC members should be held to account by people affected by decisions of the PPHCC.

Not at all 0 1 2 3 4 5 6 7 8 9 10 Strongly

15. I am open to criticism of my decisions in the PPHCC.

Never 0 1 2 3 4 5 6 7 8 9 10 Always

16. I allow people affected by PPHCC decisions to question me on why a decision was made.

Never 0 1 2 3 4 5 6 7 8 9 10 Always

17. I use pertinent information while making a decision in the PPHCC.

Never 0 1 2 3 4 5 6 7 8 9 10 Always

18. I collaborate with other ministries to achieve better health outcomes for people in my province.

Never 0 1 2 3 4 5 6 7 8 9 10 Always

19. I collaborate with the private for-profit sector to achieve better health outcomes for people in my province.

Never 0 1 2 3 4 5 6 7 8 9 10 Always

20. I collaborate with civil society and nongovernmental organizations to achieve better health outcomes for people in my province.

Never 0 1 2 3 4 5 6 7 8 9 10 Always

21. My decisions in the PPHCC are based on what I think is right rather than on evidence and analysis.

Never 0 1 2 3 4 5 6 7 8 9 10 Always

22. I believe in the power of technology in enhancing the quality of PPHCC decisions.

Not at all	0	1	2	3	4	5	6	7	8	9	10	Strongly

23. I advocate use of modern technologies in my PPHCC decision-making process.

Never	0	1	2	3	4	5	6	7	8	9	10	Always

24. I believe in measurement of the results and impact of PPHCC decisions.

Not at all	0	1	2	3	4	5	6	7	8	9	10	Strongly

25. I insist on measuring the results and impact of PPHCC decisions.

Never	0	1	2	3	4	5	6	7	8	9	10	Always

26. I oversee and ensure that PPHCC decisions made are implemented.

Never	0	1	2	3	4	5	6	7	8	9	10	Always

27. I give a higher weight to long-term interests of the society than the short-term benefits to the society while making a decision in the PPHCC.

Never	0	1	2	3	4	5	6	7	8	9	10	Always

28. I consider the concerns of poor and vulnerable people while making a decision in the PPHCC.

Never	0	1	2	3	4	5	6	7	8	9	10	Always

29. I try hard to achieve the best results with the least amount of resources when I make a decision in the PPHCC.

Never	0	1	2	3	4	5	6	7	8	9	10	Always

Scoring criteria

Questions 2 and 22 are reverse coded and receive a minus score, i.e., the score is subtracted from the total score. The maximum score that can be earned is 280.

- Score of 210 and above: Outstanding governance
- Score of 141–209: Meets most requirements
- Score of 70–140: Needs improvement
- Score below 70: Unsatisfactory governance

4. Health Governance Self-Assessment Tool for Individual Members based on TOR

Scoring guide

0%	1%–25%	26%–50%	51%–75%	76%–100%
**No activity**	**Minimal activity**	**Moderate activity**	**Significant activity**	**Optimal activity**
0%, or absolutely no activity	Greater than zero, but no more than 25% of the activity described within the ToR is met	Greater than 25%, but no more than 50% of the activity described within the ToR is met	Greater than 50%, but no more than 75% of the activity described within the ToR is met	Greater than 75% of the activity described within the ToR is met

Instrument

#	ToR	0%	1%–25%	26%–50%	51%–75%	76%–100%
**A**	**General**					
1	Knows the provincial health system's strategic purpose of community service, goals, policies, programs, services, strengths, and needs					
2	Performs the duties of PPHCC responsibly and conforms to the level of competence expected from PPHCC members					
3	Suggests possible nominees to the PPHCC or advisory bodies who are clearly women and men of achievement and distinction and who can make significant contributions to the work of the PPHCC and the provincial health system					
4	Serves in leadership positions and undertakes special assignments willingly and enthusiastically					
5	Helps establish and nurture exemplary relationships with various provincial and community stakeholder organizations and associations so as to inform the plans of the provincial health system, and also helps ensure the cost-effective implementation of those plans via influence with these other organizations					
6	Follows and stays informed about trends in the provincial health system and the PPHO and the MOPH, especially trends in public health, service quality and patient safety, health care economics, medical technologies, physician and health worker issues, regulatory frameworks for health programs, and community aspirations					
**B**	**Meeting Related**					
1	Prepares for and participates in PPHCC and subcommittee meetings, including appropriate PPHCC activities					
2	Asks timely and substantive questions at PPHCC and subcommittee meetings, while at the same time supporting the majority decision on issues decided by the PPHCC					
3	Suggests agenda items periodically for PPHCC and subcommittee meetings to ensure that significant, policy-related matters are addressed					

#	ToR	0%	1%–25%	26%–50%	51%–75%	76%–100%
**C**	**Relationship with Staff**					
1	Counsels the Provincial Public Health Director as appropriate and supports her or him through relationships with organizations or individuals, as needed					
2	Avoids asking for special favors from the staff, including special requests for extensive information, without at least prior consultation with the Provincial Public Health Director, PPHCC, or appropriate committee chairperson					
**D**	**Avoiding Conflicts**					
1	Serves the province as a whole rather than any special interest organization or constituency					
2	Avoids even the appearance of a conflict of interest that might embarrass the PPHCC or the provincial health system; discloses any possible conflicts to the PPHCC in a timely fashion					
3	Maintains independence and objectivity and does what a sense of fairness, ethics, and personal integrity require					
4	Never accepts favors or gifts from anyone who does business with the provincial health system or any of its service delivery programs or facilities					
**E**	**Fiduciary Responsibilities**					
1	Serves as a well-informed champion for public health promotion and protection, patient safety, and excellent health outcomes					
2	Carefully reads and understands the Provincial Public Health Department's financial statements and helps the PPHCC fulfill its fiduciary responsibility as an efficient provincial public health system					
**F**	**Resource Mobilization**					
1	Helps the Provincial Public Health Director implement strategies to attract diverse sources of revenue to support the provincial health system's mission through personal influence with others (e.g., donors, government agencies, etc.)					

5. **Health Governance Self-Assessment Tool for the Chair**

Scoring guide

0%	1%–25%	26%–50%	51%–75%	76%–100%
**No activity**	**Minimal activity**	**Moderate activity**	**Significant activity**	**Optimal activity**
0%, or absolutely no activity.	Greater than zero, but no more than 25% of the activity described within the ToR is met	Greater than 25%, but no more than 50% of the activity described within the ToR is met	Greater than 50%, but no more than 75% of the activity described within the ToR is met	Greater than 75% of the activity described within the ToR is met.

Instrument

#	ToR	0%	1–25%	26–50%	51–75%	76–100%
1	Leads and facilitates PPHCC meetings					
2	Develops meeting agendas					
3	Leads development of a strong PPHCC					
4	Sets goals and expectations for the PPHCC					
5	Encourages individual PPHCC members to take leadership					
6	Makes PPHCC development a priority					
7	Works with the Governance Subcommittee to identify skills, expertise, and attributes needed for the PPHCC					
8	Works with the Governance Subcommittee and individual members to recruit new PPHCC members					
9	Ensures regular opportunities for PPHCC self-assessment					
10	Oversees comprehensive PPHCC assessment every 2–3 years					
11	Assesses results of PPHCC self-assessment and considers improvements in consultation with the Governance Subcommittee					
12	Solicits inputs from all PPHCC members and ensures all PPHCC members contribute appropriately					
13	Keeps all PPHCC members engaged in the work of the PPHCC					
14	Speaks on behalf of the PPHCC regularly and especially when controversy or conflict arises					
15	Guides the PPHCC in approving plans and overseeing their implementation					
16	Facilitates PPHCC involvement in strategic					

#	ToR	0%	1–25%	26–50%	51–75%	76–100%
	planning					
17	Shares appropriate information to keep the PPHCC informed and educated					
18	Coordinates overall resource mobilization effort					
19	Reviews committee work					
20	Resolves conflicts among PPHCC members					
